# An In Vitro Investigation of the Impact of Ultrasound Induced‐Streaming Motions on the Flow Inside Lateral Features of the Root Canal

**DOI:** 10.1111/iej.70057

**Published:** 2025-10-24

**Authors:** Anastasios Koulogiannis, Anthony Damien Walmsley, Panagiota Angeli, Stavroula Balabani

**Affiliations:** ^1^ FluME, Department of Mechanical Engineering University College London (UCL) London UK; ^2^ School of Dentistry University of Birmingham Birmingham UK; ^3^ ThAMes, Department of Chemical Engineering University College London (UCL) London UK; ^4^ Hawkes Institute, University College London (UCL) London UK

**Keywords:** lateral canal, microstreaming, particle image velocimetry, root canal, ultrasonic irrigation

## Abstract

**Aim:**

The study aims to experimentally characterize the flow inside lateral canals during ultrasonic irrigation. The effect of the canal angle as well as the relative position of the endodontic file with the inlet of the channel was quantified.

**Methodology:**

An idealized endodontic geometry was 3D printed and used as a mould to fabricate a channel made of PEG‐modified hydrophilic PDMS. The mould comprises a 20 mm tapered (6%) root canal with a 0.4 mm apical diameter and a 5 mm long lateral canal with a 200 μm square cross section, forming an angle of either 90° or 60° with the axis of the root canal and placed 6 mm from the apex. The channel was filled with a NaI‐water solution and seeded with 1 μm particles. An ultrasonic instrument with a 15# K‐file was employed for the irrigation. The flow field was characterized by Particle Image Velocimetry.

**Results:**

The flow inside the lateral canals is notably affected by the position of the endodontic file. There is a competition between the impingement of the ultrasound‐induced jets on the walls of the root canal and the recirculation flows around the file, leading to either flow into the lateral canal or suction phenomena that generate backflow towards the root canal. Aligning the jet with the axis of the lateral canal maximizes the magnitude of the velocities that can be achieved therein. This alignment can only be achieved in the 90° channel. Inclining the lateral canal to 60° leads to lower velocities and a domination of the backflows towards the root canal. In both cases, a recirculation zone forms near the inlet of the channel where transverse velocities are comparable with the axial ones. The recirculation zone length is affected by the inclination of the channel. Further inside the canal, the flow becomes uniaxial.

**Conclusion:**

The flow during ultrasonic irrigation inside lateral features of the root canal is complex and considerably affected by the position of the endodontic file and the inclination of the channel. An optimum location of the file tip leads to maximization of the induced velocity inside the lateral canal, suggesting more effective cleaning.

## 1 Introduction

Root canal systems are susceptible to bacterial infections. Bacterial species living in oral cavities can gain access into confined endodontic spaces by producing acids that can break through the enamel or by moving through exposed dentinal tubules (Zehnder and Belibasakis 2015). The bacteria can adhere to hard tissue surfaces and produce extracellular polymeric substances forming a gel‐like structure called bacterial biofilm (Jhajharia et al. 2015). The presence of biofilm within the root canal may lead to dental pulp inflammation with subsequent infection and pain (Gedif Meseret 2021; Jhajharia et al. 2015; Tinanoff and Matsumoto 2014).

Root canal instrumentation plays a critical role in endodontic treatment by mechanically removing infected dentine and reducing the bacterial load within the canal system. In addition to shaping the canal for obturation, instrumentation is essential for creating space that allows irrigants to penetrate deeper into anatomical complexities such as lateral canals, isthmuses, and dentinal tubules, where bacteria often persist. However, instrumentation alone cannot eliminate biofilms due to the intricate canal morphology, which is why it must be complemented by effective irrigation and disinfection protocols (Peters et al. 2001; De‐Deus et al. 2011).

Whilst syringe‐based irrigation has been primarily used previously, ultrasonic irrigation is an alternative and more efficient approach to the elimination of bacterial biofilms (Gomes et al. 2023; Layton et al. 2015; Li et al. 2019). The main challenge in ultrasonic irrigation is the geometrical complexity of the root canal that limits irrigant penetration (Paiva et al. 2024). However, the ultrasound‐induced acoustic flows have the capacity to move into inaccessible areas such as isthmuses and lateral canals (Robinson et al. 2018). Thus, understanding the mechanics behind ultrasonic irrigation can be an important tool for the optimization of treatment protocols for effective biofilm eradication.

Extensive computational (Boutsioukis et al. 2022; Verhaagen et al. 2014) and experimental (Koulogiannis et al. 2024; Layton et al. 2015) research has been conducted on the fluid dynamics of ultrasonic irrigation inside straight and curved root canals. The effect of cavitation on biofilm removal from the root canal walls has also been quantified (Macedo, Verhaagen, et al. 2014). The effect of ultrasonic irrigation on biofilm removal from lateral features of the root canal has also been investigated employing both natural biofilms and biofilm‐mimicking hydrogels and imaging methods, such as standard (Park et al. 2023), high‐speed imaging (Macedo, Robinson, et al. 2014), confocal scanning laser microscopy (Pereira et al. 2021), and optical coherence tomography (Retsas et al. 2022).

However, studies on the impact of the ultrasound‐induced streaming motions into the flow inside these lateral features are rather limited. Robinson et al. (2018) studied the effect of irrigant properties and file characteristics on cavitation and microstreaming during ultrasonic irrigation, while Su et al. (2021) studied the extent of microstreaming into lateral canals during laser‐activated irrigation.

The present study aims to provide insights on the flow characteristics inside the lateral canals generated by the complex microstreaming phenomena around an oscillating endodontic file during ultrasonic irrigation. Given the complexity of the flow, five different positions of the endodontic file have been studied to provide a better understanding of how the generated acoustic flow field can be manipulated. Such investigations may lead to higher velocities being generated inside these lateral canals, enhancing biofilm disruption and leading to more effective cleaning. A clinically relevant endodontic geometry has been chosen that considers both morphological characteristics (inclination of the lateral canal) and material properties (hydrophilicity of dentine) of the endodontic systems, leading to a more realistic simulation of the irrigation process.

## 2 Materials and Methods

**FIGURE 1:**
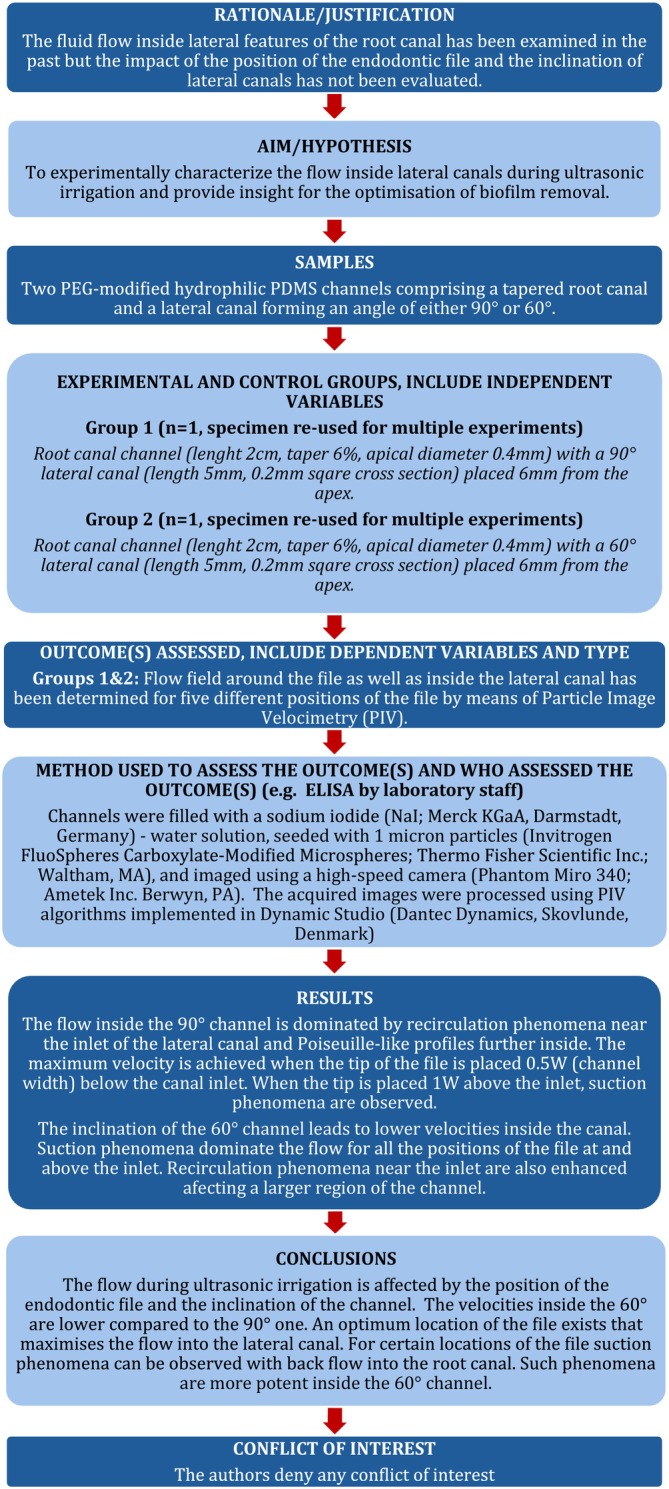
PRILE 2021 flow chart for the present study.

The manuscript of this laboratory study has been written according to the Preferred Reporting Items for Laboratory Studies in Endodontology (PRILE) 2021 guidelines (see Figure 1).

### 2.1 Channel Fabrication

**FIGURE 2:**
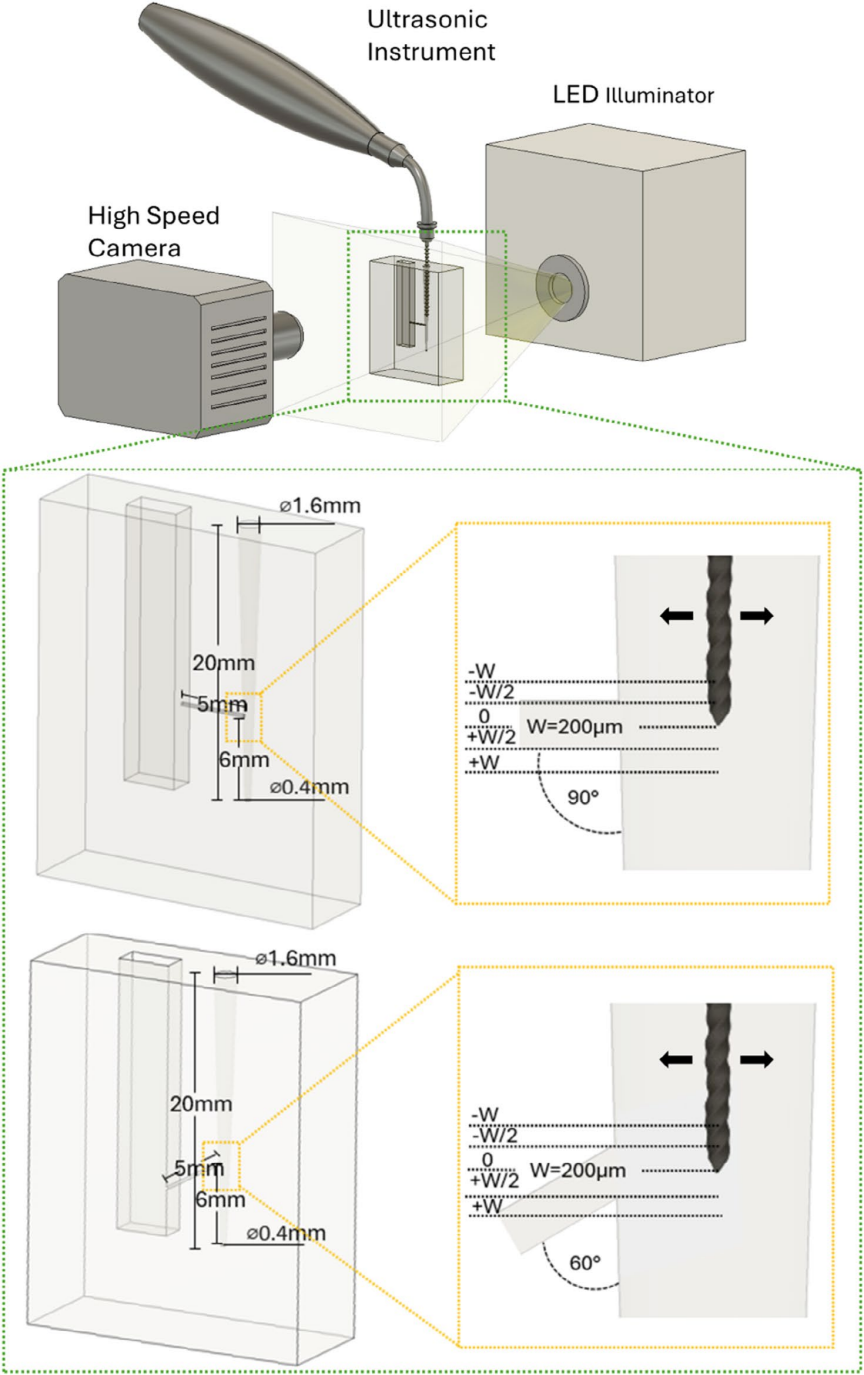
Experimental PIV setup. The inset shows the details of the two endodontic geometries as well as the different positions of the file studied. The arrows show the direction of oscillation of the file.

An idealized straight root canal geometry was selected for the experiments. The canal was 20 mm long, with 6% tapering and an apical diameter of 0.4 mm. A 5 mm long lateral canal with a 200‐μm square cross‐section was placed at 6 mm from the apical end of the root canal, forming an angle of either 90° or 60° with the principal vertical axis of the root canal (see Figure 2).

The lateral canal was connected to an open chamber having the same height as the root canal to maintain a pressure equilibrium during the experiments. The selected design was halved, and the two parts were 3D printed on a flat substrate using a resin printer (Saturn 3 Ultra; Elegoo Inc.; Shenzhen, China) and used as casting moulds to fabricate the root canal channel. To mimic the dentine properties, PEG‐modified hydrophilic PDMS was used for the casting. A mixture of 0.5% dimethylsiloxane block copolymer (DBE‐411; Gelest Inc., Morrisville, PA) and 95.5% polydimethylsiloxane (PDMS) (Sylgard 184; Dow, Midland, MI) was used, leading to a contact angle with water of 60° (Bai and Katz 2014). Prior to the casting, the 3D printed geometry was coated with a cure inhibition reducer (Inhibit X; Bentley Advanced Materials; Feltham, UK) to prevent PDMS curing inhibition. The two PDMS parts were then aligned under a microscope and clamped together using a custom‐made clamping system.

### 2.2 Ultrasonic Irrigation

For the irrigation, an endodontic tip with a #15 K‐file was attached to an ultrasonic instrument operating at 28 kHz (DTE S6; Woodpecker Medical Instrument Co. Ltd., Guilin, China). The file was inserted inside the root canal and a micrometre stage setup was used for alignment and positioning. The ultrasonic instrument was set to a fixed medium power setting of 7 out of 15 in all measurements reported in this study. This corresponds to a maximum amplitude of oscillation (in water) of about 20 μm at the tip of the file extracted from high‐speed imaging. Five positions of the tip of the file were studied in the range of −W (−200 μm) to +W (+200 μm) from the centreline of the lateral canal, where W corresponds to the width of the lateral canal (see Figure 2).

### 2.3 Particle Image Velocimetry (PIV)

A schematic of the experimental setup is shown in Figure 2. Both the root canal and the pressure chamber were filled with a 0.425 w/w sodium iodide (NaI; Merck KGaA, Darmstadt, Germany)—degassed water solution to match the refractive index of PDMS (RI of 1.42) while keeping the physical properties close to the commonly used irrigants. The solution had a viscosity of 1.67 mPa·s and a density of 1.48 g/cm^3^ at 20°C. The flow was seeded with 1‐μm particles (Invitrogen FluoSpheres Carboxylate‐Modified Microspheres; Thermo Fisher Scientific Inc.; Waltham, MA), illuminated using a white LED light and imaged using a high‐speed camera (Phantom Miro 340; Ametek Inc., Berwyn, PA) operating in a single‐frame mode. 1000 images were acquired at a frame rate between 1 and 6 kHz selected based on the maximum particle displacement in each case.

The pixel intensity values of the acquired images were then inverted and the background was subtracted. The processed images were cross‐correlated using an adaptive PIV algorithm, implemented in Dynamic Studio (Dantec Dynamics, Skovlunde, Denmark), to obtain the instantaneous velocity fields, and the results were time‐averaged. The term instantaneous velocity field, as used here, refers to the flow field captured at a given time point and does not imply fully resolving the oscillatory nature of the flow induced by the ultrasonic instrument. An interrogation window size of 16 × 16 pixels was employed, and a 3‐iteration‐3 × 3 moving average validation filter with a 0.1 acceptance factor was applied to the generated vector fields. A 3 × 3 neighbouring vector average filter was then used to smoothen the results.

Two optical configurations were used depending on the region of interest. For measurements near the oscillating file, a 10× Infinity‐Corrected Long Working Distance (WD) objective was used, providing a spatial resolution of ~2 μm/pixel and a depth of field of ~3.2 μm. For measurements inside the lateral canal, a 20× objective has been used leading to a resolution of ~0.7 μm/pixel and a depth of field of ~1.6 μm. Tracer particle concentration was manually adjusted to maintain approximately 10–15 particles per interrogation window. It should be noted that while fluorescent particles were used, a shadowgraphy (shadow‐PIV) method was employed in the present study.

The 2D shear stress distribution was estimated from the measured velocity fields using:

(1) $$\tau_{\mathrm{xy}}=\mu \left(\frac{\partial v}{\partial x}+\frac{\partial u}{\partial y}\right)$$

where μ is the dynamic viscosity of the solution and *u* and *v* are the velocity components on the *x* and *y* axes respectively. A central differencing scheme throughout the domain, and a forward or backward differencing scheme along the edges were used to calculate the derivatives.

## 3 Results

**FIGURE 3:**
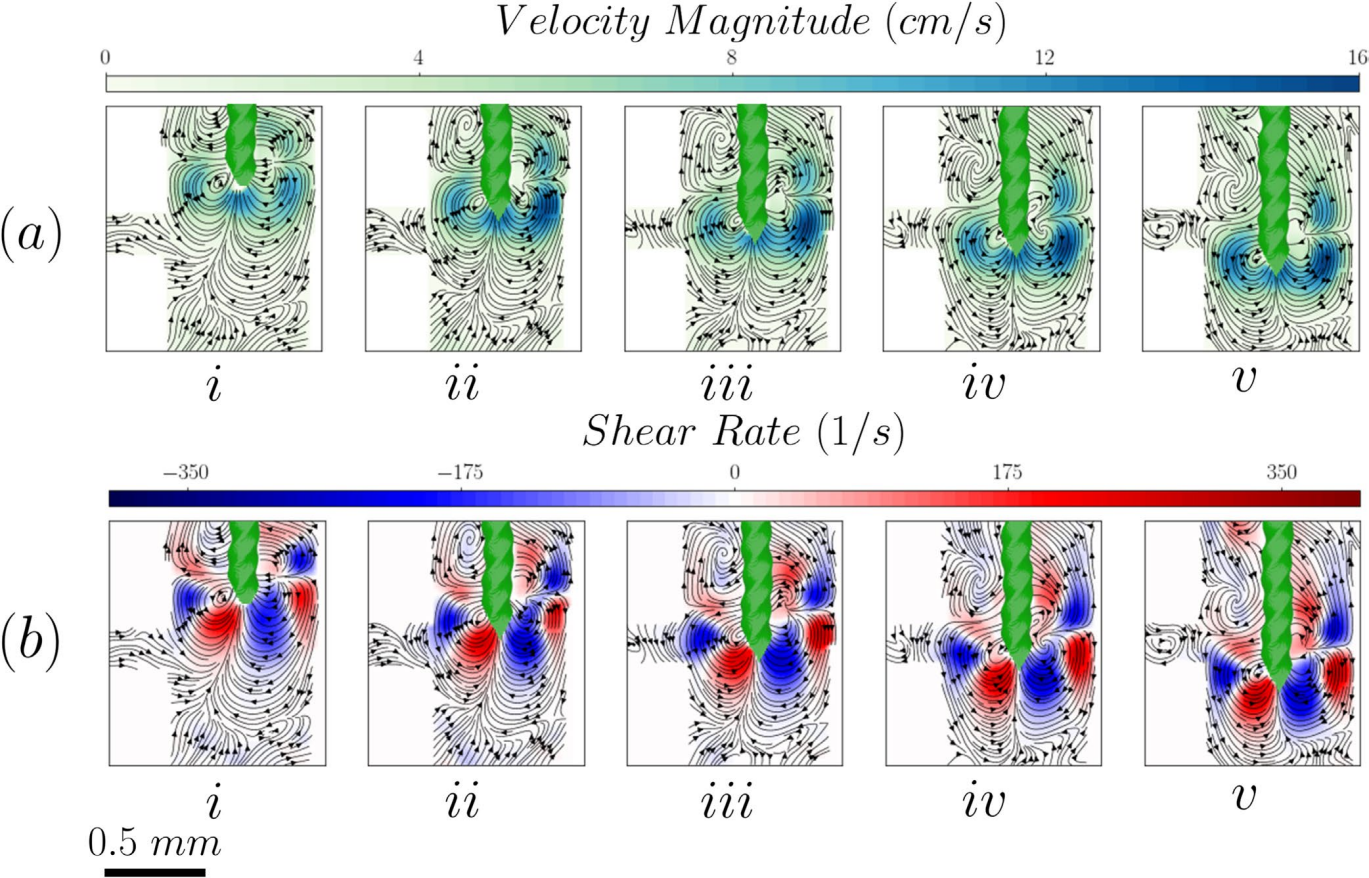
Velocity streamlines, magnitude (Panel a) and shear rate (Panel b) distributions inside a root canal with a 90° lateral canal for different file positions. (i to v indicate the file tip location: i: −W, ii: −W/2, iii: 0, iv: +W/2, v: +W).

Time‐averaged velocity and shear rate maps, with superimposed streamlines of the flow around the tip of the endodontic file, are shown in Figure 3 for the 90‐degree lateral canal and the five positions studied therein.

The oscillation of the file gives rise to two jets generated slightly above the tip of the file at about one file diameter (150 μm). Their impingement on the walls of the root canal generates four vortical structures and leads to high velocity and shear rate regions in the area around the file. The symmetry of these flow structures is affected by the position of the file in relation to the lateral canal, which causes a change in the boundary condition (and hence the degree of confinement the flow experiences) on the left side of the root canal. Thus, when the file is placed 0.5 W to 1 W above the entrance of the side channel (see Figure 3a‐i,ii), both jets impinge on the walls of the root canal, leading to the symmetry of the vortices. As the file moves deeper into the root canal by 0.5 W (see Figure 3a‐iii), part of one of the jets penetrates the lateral canal. This penetration partially disrupts the lower‐left vortex, breaking the symmetry of the flow field. Further advancement of the file affects the alignment between the jet axis and the lateral canal. Consequently, the upper‐left vortex starts to fade away and the lower‐left vortex strengthens (see Figure 3a‐iv,v). A similar trend can be observed in the shear rate distribution. While the tip of the file remains 1 W to 0.5 W higher than the entrance of the lateral canal, the high shear regions are located primarily near the upper walls of the root canal and around the file tip (see Figure 3b‐i,ii). As the file is moved downwards by 0.5 W to 1 W (see Figure 3b‐iii,iv), the high shear rate regions coincide with the lateral canal inlet and move below it as soon as the file reaches its lowest position, namely 1 W below the inlet of the lateral canal (see Figure 3b‐v).

**FIGURE 4:**
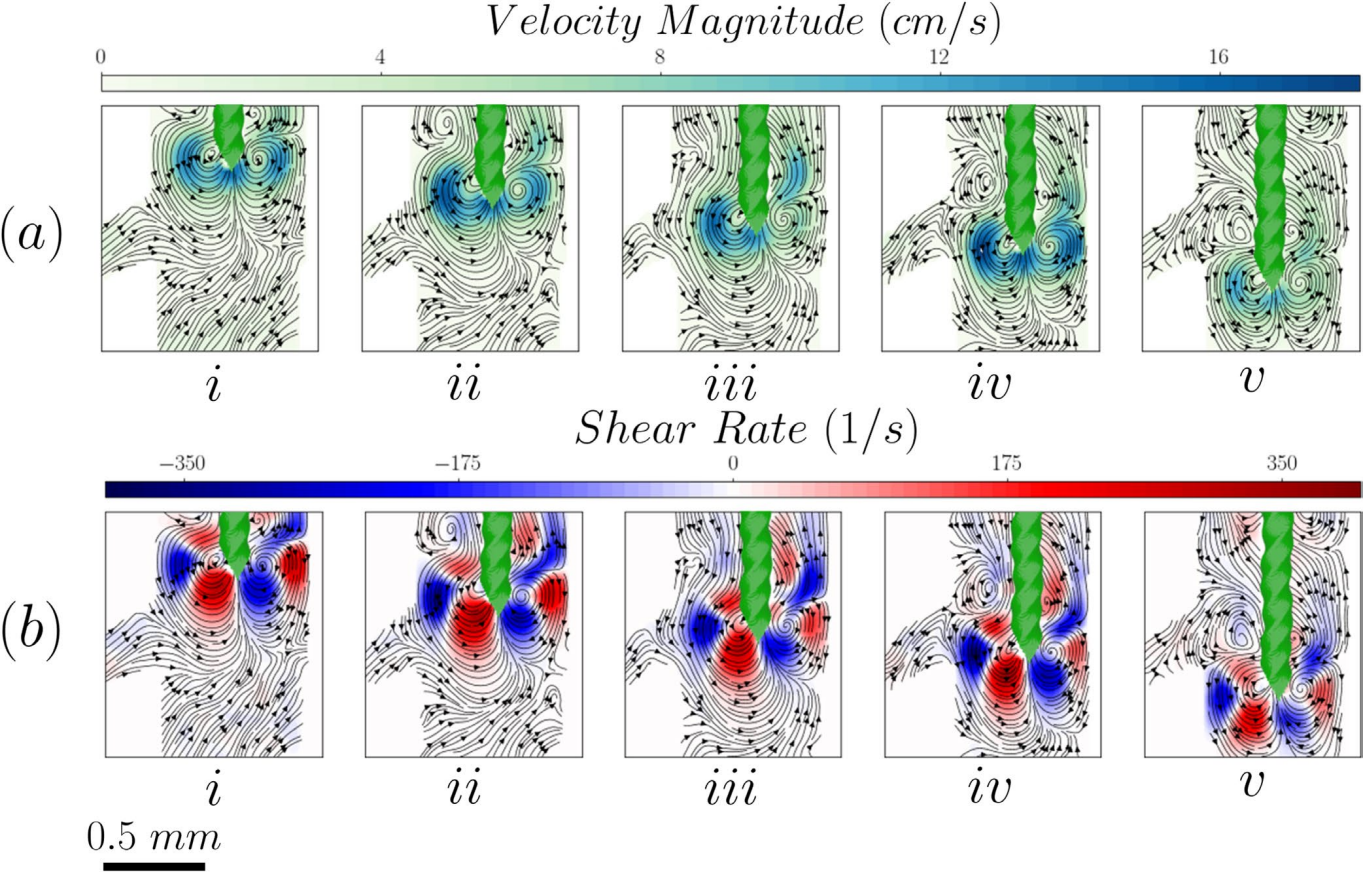
Velocity streamlines, magnitude (Panel a) and shear rate (Panel b) distributions inside a root canal with a 60° lateral canal for different file positions. (i to v indicate the file tip location: i: −W, ii: −W/2, iii: 0, iv: +W/2, v: +W).

The results in Figure 4 show that the lateral canal angle does not notably affect the velocity and shear rate magnitude around the file. There is, however, an effect on the flow structures that can be attributed to the change of the boundary condition on the left side of the root canal wall.

When the file tip is at a distance of 1 W above the entrance of the side channel (see Figure 4a‐i), the four vortical structures can be observed again. However, as the file moves deeper inside the root canal, the inclination of the lateral canal forces the jet to impinge at an obtuse angle to the opposite wall of the lateral canal. This strengthens the recirculatory flow below the tip of the file while weakening the two upper vortical structures (see Figure 4a‐ii,iii). As the file is moved further downwards to 0.5 W and to 1 W, this effect is dampened and the two upper vortices reappear (see Figure 4a‐iv,v). This change in the boundary condition does not appear to affect the shear stresses as their magnitude and distribution around the tip of the file is similar for the five different positions (see Figure 4b‐i,v).

**FIGURE 5:**
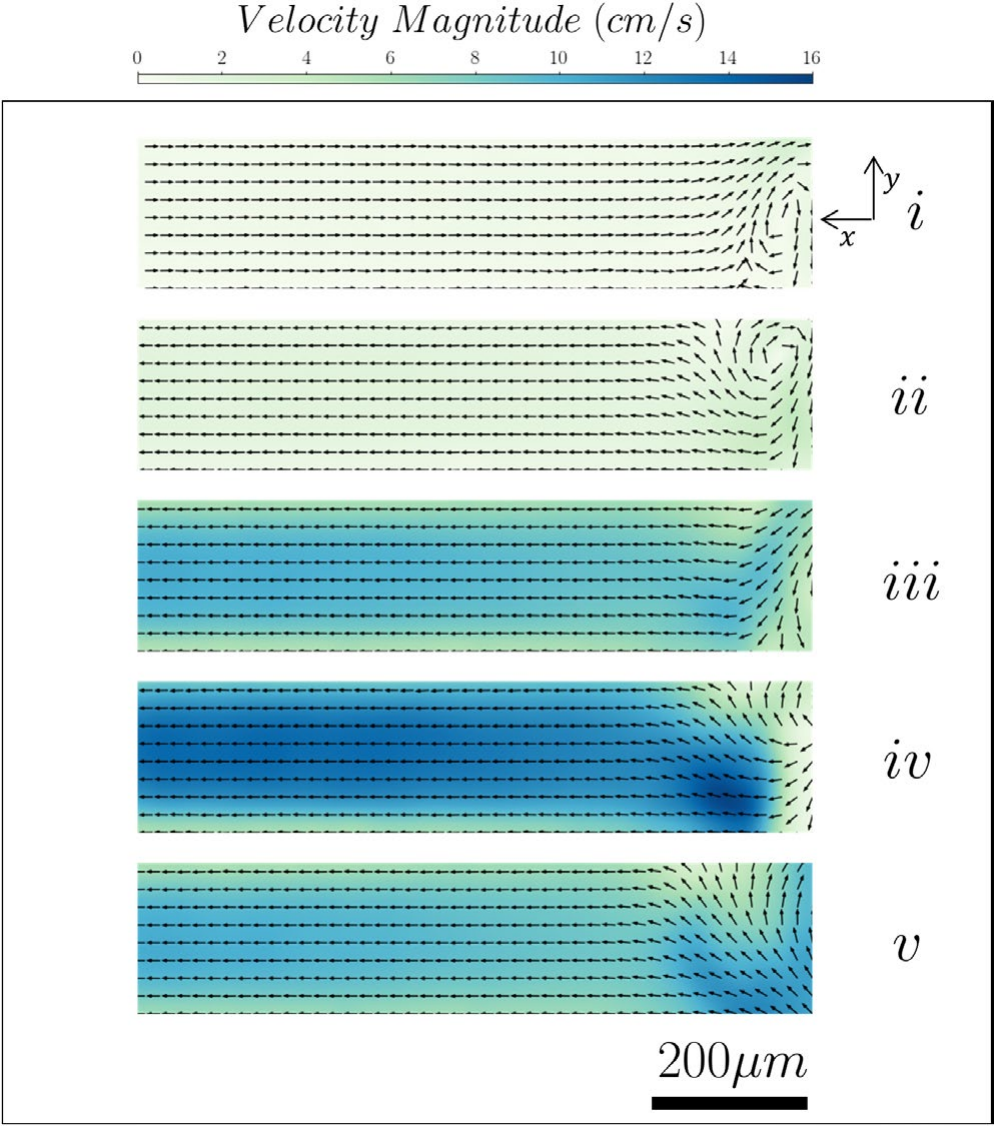
Velocity vectors and magnitude contours inside a 90° lateral canal.

**FIGURE 6:**
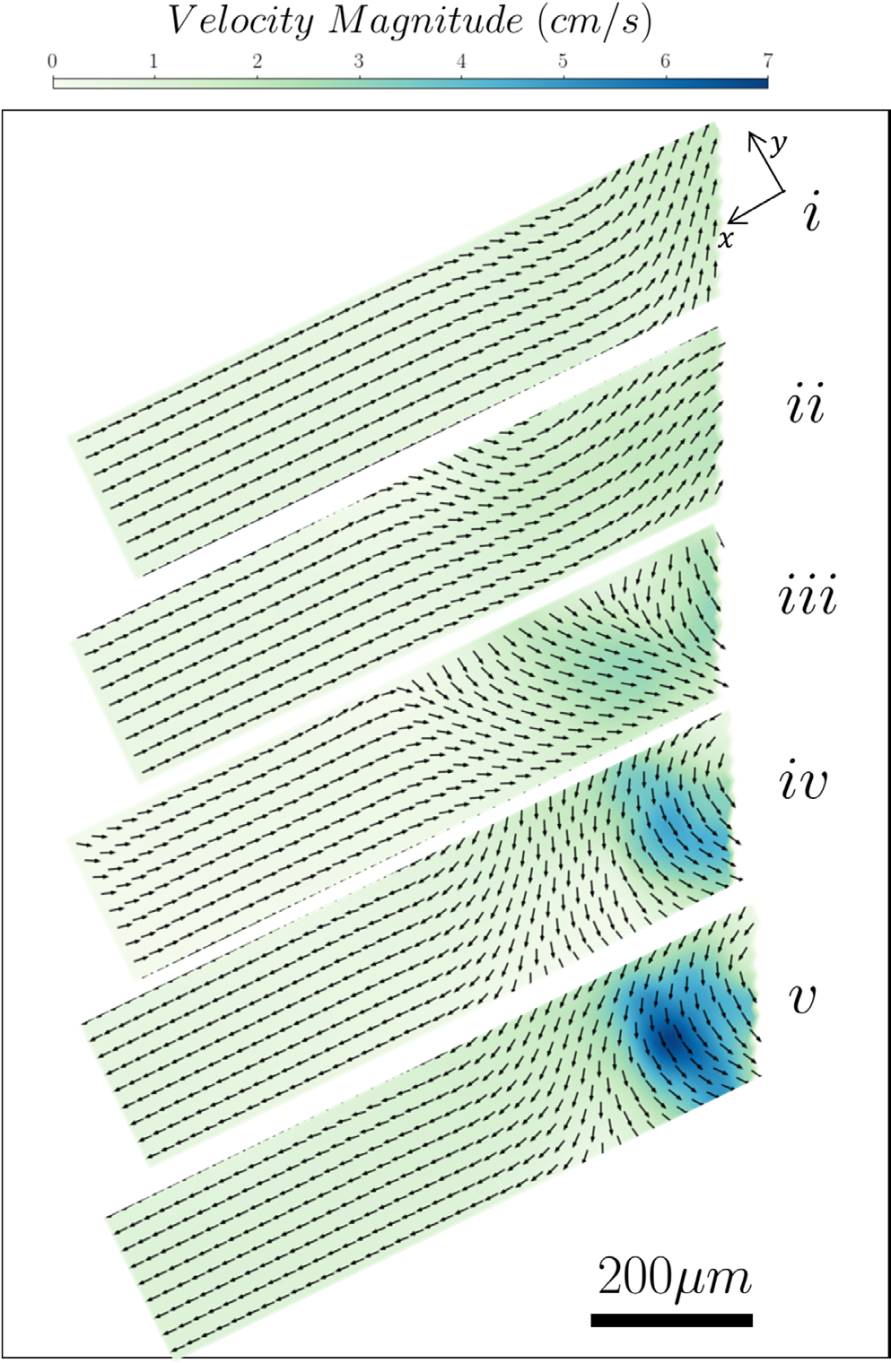
Velocity vectors and magnitude contours inside a 60° lateral canal.

The time‐averaged flow field inside the 90° and 60° lateral channels for the five different file positions can be seen in Figures 5 and 6 respectively.

The flow field inside the 90° channel is markedly affected by the position of the file. When the tip is placed 1 W above the inlet of the channel (see Figure 5i), the recirculatory flow developed below the file induces a suction phenomenon inside the lateral canal. The flow field is dominated by a backflow towards the root canal, while a recirculation area can be observed near the inlet of the channel. As the file is moved downwards by 0.5 W (see Figure 5ii), there is a reversal in the direction of the flow; a forward flow towards the outlet of the channel is evident and is accompanied by a small recirculation flow region at the upper wall near the inlet. Alignment of the file tip with the centreline of the lateral canal (see Figure 5iii) leads to partial penetration of the jet inside the channel; this induces a flow into the lateral canal characterized by relatively high magnitudes of velocities and results in the disappearance of the recirculation region observed previously near the inlet. A similar behavior can be observed when the tip is placed 0.5 W to 1 W below the channel (see Figure 5iv,v). However, the direction of the jet flow into the lateral channel is opposite to that induced in the previous location in Figure 5iii. The magnitude of the induced flow velocities in the lateral canal reaches a maximum when the tip is placed 0.5 W below the channel inlet (see Figure 5iv), and the jet is almost perfectly aligned with the axis of the lateral canal. The flow field inside the 60° inclined channel exhibits similar characteristics, albeit moderated by the angle between the acoustic jet and the axis of the lateral canal. When the file tip is located higher or at the same height as the channel inlet (see Figure 6i–iii), the induced vortical structures around the tip lead to suction of the fluid inside the channel, creating a back flow with relatively low velocities. When the tip is moved 0.5 W to 1 W below the lateral channel inlet (see Figure 6iv,v), the flow direction reverses and a forward flow into the lateral canal is observed. The velocities, however, remain low compared to those in the 90° channel. A recirculation region with higher velocities can be observed near the inlet of the lateral canal.

It is important to note that the velocity fields reported in Figures 3, 4, 5, 6 are based on separate experiments using different tracer particle sizes based on the size of the investigated regions. For measurements near the file and within the main root canal (Figures 3 and 4), 20 μm tracer particles were used. These larger particles were selected to improve visualization of the larger field of view but were unable to reliably enter or resolve the flow field inside the narrower lateral canal. However, the measurements taken in the interior of the lateral canal (Figures 5 and 6) utilized 1 μm particles to properly resolve the flow therein. As such, Figures 3 and 4 illustrate the mechanisms generating the flow around and inside the lateral canal, while Figures 5 and 6 provide a more detailed representation of the flow within the canal. These datasets are therefore complementary and not intended to be directly compared.

**FIGURE 7:**
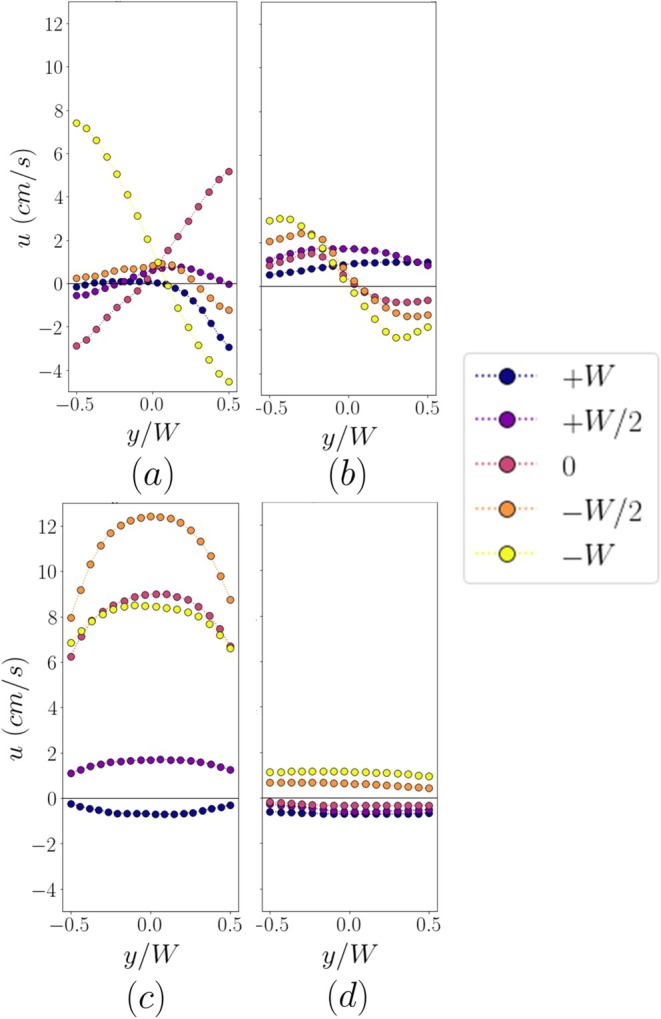
Axial velocity across the cross section at the inlet (a, b) and mid‐length (c, d) of the lateral canal of the 90° and 60° channel, respectively.

The profiles of the axial velocity across the inlet and a cross section mid‐length of the lateral canal (∼2.5 widths from the inlet) are shown in Figure 7 for the five file positions in the 90° and 60° channels. The axial velocity profiles at the inlet of the 90° channel (Figure 7a) appear skewed, and the degree of this skewness varies with the location of the file. Negative velocities are observed in part of the cross section, reflecting the flow reversal and recirculation regions observed in Figure 5. The velocity magnitude remains low in most locations, except when the tip location is aligned with the axis or the lower wall of the lateral canal (Figure 5iv). The corresponding profiles are highly skewed on opposite sides to each other, exhibiting both negative and positive velocities on either side of the channel centerline.

Higher velocities with a parabolic profile are observed mid‐length of the lateral canal (see Figure 7c). Velocities are negative when the tip is placed 1 W above the channel inlet because of the observed suction phenomena, while the highest velocities are achieved when the file is placed 0.5 W below the inlet.

The axial velocity profiles exhibit different behavior in the 60° lateral channel. The inlet velocity magnitude (Figure 7b) increases as the file tip is moved below the inlet of the channel, and the profiles are highly skewed in all these locations. Nevertheless, the maximum velocity achieved (∼3 cm/s) is four times lower compared to the 90° channel (Figure 7a). Axial velocities remain low at the mid‐length of the canal. At the three locations at and above the channel inlet, the axial velocities consistently remain negative, indicating the aforementioned suction phenomena.

**FIGURE 8:**
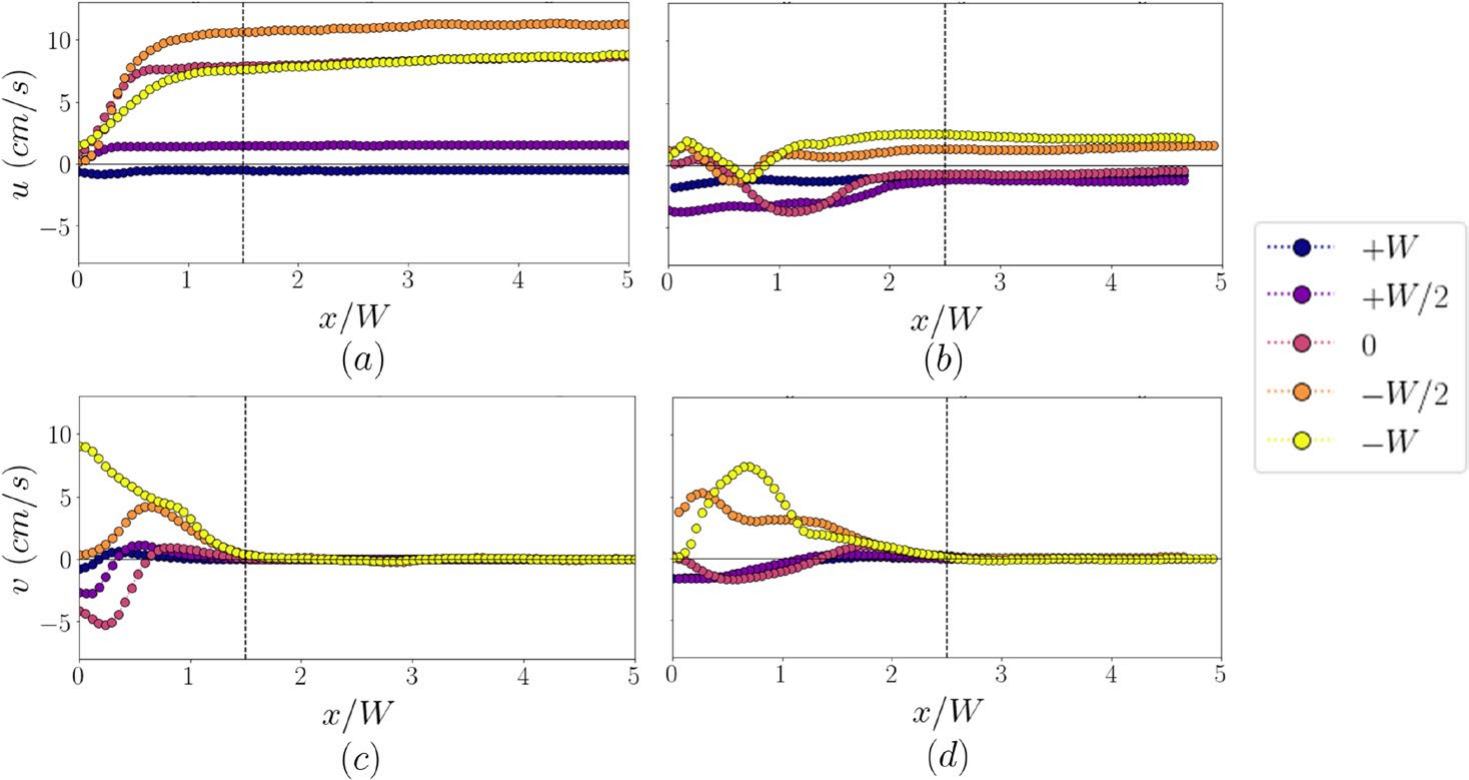
Average axial (a, b) and transverse velocity (c, d) along the 90° and 60° channels respectively.

The variation of the average axial and transverse velocity magnitudes along the lateral canals is shown in Figure 8. The profiles were calculated by averaging the axial and transverse velocities across the width of the channel for different positions along the channel for a total length of five widths.

Near the inlet of the 90° channel (Figure 8a) and up to 1.5 W, a flow region characterized by rapidly rising axial velocities and an important transverse velocity component (Figure 8b) is evident for locations 0 to +W due to the recirculation regions and the jet impingement phenomena observed in Figure 5. Beyond that length, the flow becomes uniaxial, as evidenced by the constant axial velocities and negligible transverse component. Similar behavior can be observed in the 60° channel. A developing flow region where both axial and transverse velocities vary along the length of the channel can be observed near the inlet up to about 2.5 channel widths. This indicates that the impact of the streaming motions from the tip extends further into the lateral canal compared to the 90° channel. However, the velocities reached there remain almost an order of magnitude lower than in the 90° channel.

## 4 Discussion

The relative position of the tip of the endodontic file with respect to the lateral canal inlet plays an important role in the generated flow field. As seen in Figures 3 and 4 due to the oscillation of the file, we observe the generation of two flow phenomena: two acoustic jets perpendicular to the file impinging on the walls and hence creating two recirculation zones above and below them. The jets seem to be generated slightly higher from the tip of the file at approximately one diameter. This agrees with both computational flow studies (Boutsioukis et al. 2022) and experimental results on the hydrogel removal pattern from an isthmus (Macedo, Robinson, et al. 2014). Given that the diameter of these jets is comparable to the width of the lateral canal, small changes in the position of the file can lead to the alignment or misalignment of the jet with respect to the axis of the lateral canal. As seen in Figure 3, the jets are well aligned with the canal axis between positions −W/2 and −W, where the velocity magnitudes reach their highest values inside the lateral canals. Figure 3 shows that part of the jet impinges on the root canal wall near the inlet to the lateral canal for all these positions, apart from the top one (−W). In this case, the lateral canal inlet experiences a recirculatory flow rather than the high‐velocity jet.

A similar phenomenon is observed inside the 60° lateral canal (Figure 4). In this case, however, there is an angle between the jet and the canal axis. Therefore, even when the jet is generated at the same height as the inlet of the lateral channel, it impinges on its walls and thus its effect further inside the canal is limited. This explains the presence of recirculation regions in the flow inside the lateral canal in most cases.

A remarkable finding of this study is that for certain file locations, suction can be observed where backflow is generated towards the root canal. While flow studies inside open‐ended lateral canals have shown that flow is primarily towards the outlet (Robinson et al. 2018), in the presence of a hydrogel blocking the channel, some suction phenomena can be observed (see Sup. Information by Macedo, Robinson, et al. 2014). In the present study, a similar effect has been simulated using an outer tank and thus inducing an outlet pressure on the lateral canal. While the main reason for adding this tank was to avoid capillary‐induced flow due to the hydrophilicity of PEG‐PDMS, the pressure boundary conditions are, in addition, more effective in simulating partial blockages caused by biofilms or dead‐end lateral canals, offering more clinically relevant conditions for studying flow in endodontic systems.

It should be noted that while conservation of mass dictates that the net flow through the lateral canal should be zero in a truly steady‐state system with constant fluid levels in the open chamber and main root canal, this is not the case here where a net flow is observed in the lateral canal as the level of the free surface of the two chambers can change during the application of ultrasound. Due to the extremely low flow rates therein and short experimental durations used, these fluid level changes can be considered negligible.

The study highlighted the existence of a recirculation zone near the inlet of the channel arising from the microstreaming motions induced by the tip and their relation to the lateral canal axis/geometry. Such flow regions could lead to debris trapping and affect the successful elimination of bacteria during the irrigation process. While the length of this recirculation zone is not substantially affected by the position of the file, it generally increases with the angle of the lateral canal. This again can be attributed to the impingement of the jet on the inner walls of the 60° lateral canal.

The flow profiles shown in Figures 7 and 8 focus on the characteristics of the flow both near the inlet and further inside the lateral canal. Inside the 90° channel, a Poiseuille‐like profile with negligible transverse velocities is observed, signifying a fully developed channel flow. The flow inside the 60° channel is different, exhibiting a plug‐like velocity profile of a lower magnitude. Previous studies have focused on the flow field only in a small region near the inlet of lateral canals (Robinson et al. 2018).; understanding the effect of acoustic streaming further inside the lateral channel is equally important. Biofilms can reside over the whole length of lateral canals and thus flow characteristics, such as velocity magnitude and shear rate, can severely affect their disruption. Although the 90° canal showed higher velocities at the inlet, which can be beneficial for debridement in that region, the 60° canal shows a more plug‐like velocity profile at mid‐length instead of a parabolic one. This leads to steep velocity gradients and thus increased wall shear rates, which can also enhance debridement and may be comparable to the 90° case despite the lower velocities.

While Particle Image Velocimetry is a powerful technique for quantifying flow fields, it has some limitations, especially in the context of confined and high‐speed flows like those found in endodontic systems. Due to the high velocities and small spatial scales involved in this study, compromises had to be made between spatial resolution, temporal resolution, and field of view. Additionally, regions with strong out‐of‐plane motion or large particle displacement near the file may lead to underestimation of velocities. Interrogation window sizes and particle seeding densities were optimized as much as possible, but these factors can significantly affect the accuracy of the resulting vector fields. Furthermore, due to the frame rate of the camera employed being lower than the frequency of the ultrasonic instrument, the oscillatory components of the flow (which can lead to higher instantaneous velocities) could not be fully resolved and thus the present study focused on the steady component. These limitations are inherent to time‐resolved PIV in such microenvironments and should be considered when interpreting the results, particularly near the file tip and in high‐shear regions. However, they do not compromise the primary aim of the study, which was to characterize the broader flow patterns, particularly those affecting the flow inside the lateral canal, which in this case are far from the tip of the file. Prior work (Verhaagen et al. 2014) has shown that velocities near the file decay rapidly with distance, supporting the relevance of the flow structures captured here.

It should be noted that the file has been placed in such a way that the oscillation is along the direction of the lateral canal, which leads to the aforementioned flow structures and is known to maximize the induced flow (Jiang et al. 2010). This optimum orientation allows us to understand the flow phenomena and generalize our results but can often be impossible clinically, especially when taking into account geometrical irregularities of the root canal.

The velocity magnitude results inside the lateral canal are of the same order of magnitude as the ones reported in the existing literature (Robinson et al. 2018); however, the ones near the inlet are slightly lower. This can be attributed to differences in the irrigation parameters (file size, oscillation amplitude, etc.) as well as the temporal resolution of the imaging method used. Ultrasonic flows are known to be dominated by both acoustic streaming and cavitation. Cavitation can affect the flow field both around the file and inside the lateral canal (Robinson et al. 2018). In the present study, a degassed irrigant was used to decouple the two phenomena and focus only on the effect of acoustic streaming on the generated flow field. It should be noted that degassing removes only dissolved gases and does not eliminate the possibility of cavitation. However, no cavitation bubbles were observed during imaging, and thus cavitation is not believed to have affected the flow under the conditions of our experiments. In the presence of cavitation, velocity magnitudes are expected to be higher than the ones reported here.

The present results show that the flows around ultrasonic files are complex and depend on the orientation of the file to the side channels within a root canal. This may influence how a clinician uses such instruments during root canal treatment. It is worth noting that the anatomical variability of lateral canals must be considered when studying irrigant dynamics. While many lateral canals are closed‐ended and surrounded by periodontal tissues, studies have shown that they can also be partially patent or connected to adjacent anatomical structures, such as isthmuses or deltas (Mazzi‐Chaves et al. 2020). These features may allow some fluid transport, resulting in a pressurized but not entirely stagnant system. In this study, we introduced a pressure boundary condition at the distal end of the lateral canal to better reflect these partially closed conditions. Compared to previous models employing fully open‐ended channels (Macedo, Robinson, et al. 2014; Macedo, Verhaagen, et al. 2014; Su et al. 2021), our setup provides higher hydraulic resistance and enhances physiological relevance, thus offering a well‐controlled environment for future experimental and computational studies of irrigant transport in complex canal morphologies. The geometry of the lateral canal used in this study, and more specifically its square cross section, represents a simplification that differs from the more commonly oval or round shapes observed in natural lateral canals. This choice was primarily driven by fabrication constraints and optical access for flow visualization during the experiment. While the sharp corners of a square cross section may induce flow separation and perturbations not typically present in vivo, these effects are likely to be highly localized and limited to the immediate vicinity of the corner regions. In the central region of the canal, where flow is more fully developed, the overall behavior is expected to remain representative of physiological conditions. Nevertheless, future work will incorporate more anatomically realistic geometries to enhance understanding of flow behavior in complex lateral canal structures and generalize our findings. Further studies are required to investigate the flow in smaller lateral features of the root canal such as the dentinal tubules and to fully assess the clinical implications of such observations.

## 5 Conclusion

Both the magnitude of the velocities and the direction of the flow inside the lateral canal are impacted by the location of the file tip. An optimum location that maximizes the flow into the 90‐degree lateral canal exists at a distance of W/2 below its inlet. Suction phenomena are observed in certain positions in which a backflow into the root canal develops. A recirculation zone forms near the inlet of the lateral canal where velocities are higher compared to the rest of the lateral canal, and the flow is more chaotic. The inclination of the lateral channel to 60 degrees can lead to larger recirculation zones and lower velocity magnitudes inside the lateral canal.

## Author Contributions

A.K. contributed to conceptualization, design of the experimental setup, data collection/analysis, and writing of the original draft. S.B. and P.A. contributed to funding acquisition, provision of resources, project administration, supervision, and review/editing of the draft. A.D.W. contributed to review/editing of the draft and provided clinical insights.

## Conflicts of Interest

The authors declare no conflicts of interest.

## Data Availability

The data that support the findings of this study are available from the corresponding author upon reasonable request.
